# Immune responses in the lungs of patients with tuberculous pleural effusion without pulmonary tuberculosis

**DOI:** 10.1186/1471-2172-13-45

**Published:** 2012-08-13

**Authors:** Diana Qama, Won-Il Choi, Kun Young Kwon

**Affiliations:** 1 Department of Internal Medicine, Dongsan Hospital, Keimyung University School of Medicine, Dalseong-ro 56, Jung-gu, Daegu, 700-712, Republic of Korea; 2 Regional Hospital, Berat, Albania; 3 Department of Pathology, Dongsan Hospital, Keimyung University School of Medicine, Dalseong-ro 56, Jung-gu, Daegu, 700-712, Republic of Korea

**Keywords:** Interferon-γ, Tuberculosis, Tumor necrosis factor-α, Vascular endothelial growth factor

## Abstract

**Background:**

Tuberculous pleural effusion (TPE) is one of the most common forms of extrapulmonary tuberculosis. Because most studies of TPE focused on the pleural space, little information regarding lung parenchyma is available. We therefore aimed to investigate immune responses in the lung parenchyma of TPE patients without pulmonary tuberculosis.

**Methods:**

Patients with any evidence of pulmonary tuberculosis, either from radiologic or bacteriologic evaluation, were excluded. Bronchoalveolar lavage fluid (BALF) was collected from 10 newly diagnosed, untreated, HIV-negative TPE patients and 10 healthy controls. We analyzed T-lymphocyte subpopulations and measured 10 cytokines in BALF. Cytokine levels in BALF were standardised using urea.

**Results:**

The concentrations of interferon-γ (IFN-γ), tumor necrosis factor-α (TNF-α), vascular endothelial growth factor (VEGF), and the CD4+/CD8+ ratio of T-lymphocytes were significantly higher in TPE patients without pulmonary tuberculosis than in the controls. Of the cytokines measured in BALF, VEGF showed the highest concentration. No difference was observed in T-helper type 2 cytokines between the 2 groups.

**Conclusion:**

There were significant immune responses and increases in IFN-γ, TNF-α, and VEGF in the lung parenchyma of TPE patients without pulmonary tuberculosis. This result suggests that TPE may induce a significant immune response in lung parenchyma.

## Background

Tuberculosis (TB) is responsible for more deaths worldwide than any other curable infectious disease. The clinical manifestations of TB are determined by host immune factors in response to mycobacterium infection. Tuberculous pleural effusion (TPE) is one of the most common forms of extrapulmonary tuberculosis [1,2].

The pathophysiology of TPE has been characterized by T-cell-mediated hypersensitivity reaction to mycobacterium or antigens thereof, in the pleural space. This process involves the accumulation of protein-enriched fluid and the migration of immune cells that are generally lymphocytic, with a particular predominance of CD4+ T- cells [3]. T helper-type 1 (Th1) cytokine levels are increased at pleural effusion in patients with TPE [4,5].

Strong Th1 activity is required for successful immunity against *Mycobacterium tuberculosis.* Interferon-gamma (IFN-γ), a Th1-type cytokine, is an important factor for immune responses to TB infection.[6] Mutations or certain polymorphisms in IFN-γ receptors increase susceptibility to *M. tuberculosis* infection, and in cases in which IFN-γ cannot be produced or cannot exert its effects, TB infection is more severe and often fatal [7,8]. Tumor necrosis factor-alpha (TNF-α) has been shown to have antimycobacterial activity and promotes granuloma formation in TB patients [7,9]. TNF-α may also be responsible for the toxic syndrome and tissue necrosis accompanying TB infection [10]. Several studies have reported that TPE is a Th1-dominant environment, and that Th1 cytokines such as IFN-γ and TNF-α predominate at pleural effusions in patients with TPE [4,11,12]. Vascular endothelial growth factor (VEGF) is another cytokine involved in the immune response to TB infection. VEGF is a potent multifunctional cytokine that contributes to angiogenesis and inflammation. TNF-α, VEGF, and IFN-γ levels are increased at pleural effusions in patients with TPE, suggesting a role for these cytokines in the immune response to mycobacterium infection [12,13].

Most previous studies on TPE have focused on the pleural space. Therefore, little information is available on lung parenchyma in TPE patients without pulmonary tuberculosis. TPE patients often have systemic symptoms such as fever. Therefore, we aimed to investigate the lung parenchymal immune response in TPE patients without pulmonary tuberculosis. We hypothesized those TPE patients without pulmonary tuberculosis would have pulmonary immunological changes. We investigated the levels of various cytokines and the composition of cells in the bronchoalveolar lavage (BAL) fluids in TPE patients without pulmonary tuberculosis.

## Methods

### Study subjects

Newly diagnosed untreated patients with TPE and healthy controls were enrolled in this study, which was approved by Keimyung University Dongsan Hospital Institutional Review Board. All patients were seronegative for HIV, and none was receiving corticosteroids or other immunosuppressive drugs. None of the patients or controls had ever smoked or had a previous history of tuberculosis. TPE was diagnosed by clinical manifestations, chest radiographic findings, exudative pleural effusion showing lymphocyte predominance, absence of cancer cells, and adenosine deaminase (ADA) level (>40 IU/L) [14,15]. We performed *M. tuberculosis* polymerase chain reaction (MTB-PCR) analysis, acid-fast bacilli (AFB) smear, and AFB culture using bronchoalveolar lavage fluid (BALF), sputum, and pleural fluid in all study subjects except control. We excluded any patients who showed any evidence of pulmonary tuberculosis either from radiologic or bacteriologic evaluation. As control subjects, 9 men and 1 woman with a mean age of 45 ± 14 years volunteered to participate in the study. All volunteers had normal chest radiographic findings, no symptoms of disease, and were not taking any medication. Written informed consent was obtained from all patients and the control group prior to bronchoscopy.

### Bronchoalveolar lavage

Bronchoscopy was performed according to a standardized protocol, and BALF was collected for microbiologic and immunologic examination including mycobacterial smear and culture, biochemical and cytological analysis, and MTB-PCR and cytokine assays. In brief, a flexible bronchoscope was wedged in the right middle lobe or lingula of the left upper lobe, at the same side of the TPE while the patient was under intravenous sedation. Sterile saline (30 mL) at room temperature was instilled 5 times, and the instilled fluid was aspirated using gentle suction after each aliquot and collected into sterile polypropylene tubes. Any subject who could not tolerate the entire procedure or whose returned fluid was <60% of the total infused volume was excluded from further study. The fresh BAL fluid samples were immediately stored at 4°C, and samples were sent to the relevant diagnostic laboratories. The pooled BAL fluid was centrifuged at 800 *g* for 10 min. The supernatant fluid was rapidly frozen and stored at −80°C prior to use. PCR testing for *M. tuberculosis* complex was performed using the AMPLICOR kits (Roche Diagnostics, Mannheim, Germany) for respiratory specimen preparation, amplification, and detection with the COBAS AMPLICOR analyzer (Roche Diagnostics, Basel, Switzerland) according to the manufacturer’s instructions. Differential counts were performed on Wright-Giemsa-stained cytocentrifuge preparations, which were made with a cytospin. The cell pellets were resuspended in RPMI 1649 medium and used for lymphocyte subset analysis by flow cytometry (FACScan; Becton Dickinson, Mountain View, USA). Bronchoscopy was performed on all patients before they commenced antituberculous chemotherapy. In the control group, BALF collection was performed in the right middle lobe bronchus.

### Cytokine assay

Assays of cytokines in BALF were performed using a Bio-Plex human cytokine 10-plex assay kit (Bio-Rad, USA) according to the manufacturer’s instructions. Bio-Plex cytokine assays are multiplex bead-based assays involving diverse matrices that are designed to quantify multiple cytokines. The 10-plex assay kit contains beads conjugated to monoclonal antibodies specific for interleukin (IL)-1-alpha (IL-1α); IL-1-beta (IL-1β); IL-2, -4, -6, -8, and −10; TNF-α; INF-γ; and VEGF. The frozen aliquots of BALF were thawed to room temperature prior to each assay. Standard curves and the concentration of cytokines within samples were generated with the Bio-Plex Manager Software (Bio-Rad, USA).

### Standardization of cytokine concentrations in BAL fluid (BALF) by using urea

The standardization of cytokine levels in the BALF is based on the concept that aliquots of sterile normal saline infused through the bronchoscope mix with epithelial lining fluid (ELF). When the saline is recovered by aspiration, the ELF and its components are recovered along with it. BALF cytokine concentration was adjusted using urea.[10,16] The BAL cytokine concentration was determined after correction of dilution factor as ratio of serum urea/BAL urea [10,16].

### Statistical analysis

All values were expressed as means ± standard deviation. Data were analysed by SPSS version 18 (SPSS Inc., USA). A *χ*^*2*^ test was used to compare frequencies. The Wilcoxon signed-rank sum test was used for the comparison. Bonferroni correction was performed in the multiple comparisons. Differences were considered significant at *P* < 0.05.

## Results

### Basic information and results of examinations

During the study period, 12 TPE patients were enrolled; of these, 1 patient showed AFB culture-positive BAL, and 1 patient showed MTB-PCR-positive BAL. These 2 patients were subsequently excluded. We therefore analysed 6 female and 4 male TPE patients. In the pleural fluid microbiologic study, 1 patient showed MTB-PCR-positive, and another 1 patient showed AFB culture-positive. The mean age of the TPE patients was 48 ± 21 years. Pleural effusions were unilateral in all patients. Five patients (50%) had right-side pleural effusions, and 5 (50%) had left-side pleural effusions.

In the TPE group, 5 patients (50%) yielded positive tuberculin skin tests (>10 mm) and 6 patients (60%) had fever. All TPE patients finished the short course of tuberculosis treatment without treatment-failure or side effects. Pleural thickening was observed 6 months after finishing treatment in 1 patient. There was no significant difference in mean age between the TPE and control groups.

### Differential cells recovered from BALF

The BALF differential cell counts in TPE patients compared to the controls are presented in Table 1. The ratio of CD4+/CD8+ T lymphocytes was significantly increased in the BALF of TPE patients than in that of the controls.

**Table 1:** Bronchoalveolar lavage fluid differential cell counts in the tuberculous pleural effusion (TPE) and control groups

	**TPE group**	**Control group**	***P*value**
CD4/CD8+ ratio	2.8 ± 1.14	1.56 ± 0.82	0.002
Alveolar macrophage (%)	85.3 ± 14	91.3 ± 5.03	NS
Lymphocytes (%)	12 ± 10.6	7.45 ± 5.26	NS
Neutrophils (%)	2.55 ± 4.09	0.8 ± 0.71	NS
Eosinophils (%)	0.25 ± 0.35	0.5 ± 0.85	NS
Cell number (10^4^/mL)	10 ± 12	12 ± 9	NS

Data represent mean ± SD; NS = not significant.

* Bonferroni correction p < 0.0083.

### Levels of cytokines in BALF

The IFN-γ, TNF-α, and VEGF levels in BALF were significantly higher in TPE patients than in the controls (Table 2). The levels of all other cytokines, except IL-10, measured were higher in the TPE group than in the control group.

**Table 2:** Cytokine levels in bronchoalveolar lavage fluid

	**TPE group**	**Control group**	**P value**
IFN-γ	17.36±17.26	1.43±1.95	0.0045*
TNF-α	18.92±15.04	4.24±4.29	0.004*
IL-1α	16.37±23.44	4.74±3.83	0.069
IL-1β	22.45±31.76	2.78±3.62	0.035
IL-2	6.26±19.79	0.74±1.68	0.195
IL-4	34.16±46.72	8.17±11.23	0.052
IL-6	5.78±18.27	0.94±2.08	0.207
IL-8	132.73±280.57	8.68±11.45	0.089
IL-10	17.63±29.55	22.24±25.79	0.357
VEGF	115.28±99.21	3.709±5.349	0.001*

TPE = tuberculous pleural effusion; values represent mean ± SD; data after standardization shown as picograms per millilitre of epithelial lining fluid (ELF) (pg/mL ELF; IFN-γ = interferon gamma; TNF-α = tumor necrosis factor-alpha; IL-1α = interleukin-1-alpha; IL-1β = interleukin 1-beta; IL-2 = interleukin-2; IL-4 = interleukin-4; IL-6 = interleukin-6; IL-8 = interleukin-8; IL-10 = interleukin-10; VEGF = vascular endothelial growth factor).

* Bonferroni correction p < 0.005.

## Discussion

In the present study, we found that BALF from TPE patients without pulmonary tuberculosis contained increased levels of IFN-γ, TNF-α, and VEGF. Moreover, we found an increase in the CD4+/CD8+ ratio of T lymphocytes in BALF from the TPE patients.

We measured the TNF-α level in BALF of newly diagnosed, untreated TPE patients without pulmonary tuberculosis. The TNF-α level was nearly 5-fold higher in TPE patients than in the controls. In previous studies, TNF-α level has been found to be higher in BALF in patients with pulmonary TB than that in healthy controls [10,17]. Other studies demonstrated that TNF-α level in BALF in smear-negative active pulmonary TB patients were 4.4-fold higher than that in the controls [18], and higher TNF-α mRNA expression levels in the lungs of mice with latent tuberculosis infection (LTBI) compared to the controls [19]. Thus, the increase in TNF-α levels in BALF observed in the present study could result from an inflammatory reaction in response to tuberculosis infection.

VEGF is an angiogenesis and inflammatory mediator, which has been associated with TB infection activity,[13,20] and levels decline after successful TB treatment [21]. In our study, we found that VEGF levels in BALF from patients with TPE without pulmonary tuberculosis were 31-fold higher than that in the controls. In previous studies, VEGF was measured in serum or in pleural effusion among TB patients [12,21]. VEGF levels in serum were approximately 2–4 times higher in patients with active pulmonary TB than in the controls [21,22], and in pleural effusion, it was approximately 15–39 times higher in patients with TPE compared to transudates secondary to congestive heart failure [12,13]. Another study demonstrated that VEGF levels were higher in LTBI patients than in the controls, and VEGF measurement was one of the markers used to discriminate LTBI from the controls [23]. As for TNF-α, we suggest that an increase in VEGF levels in BALF indicates an inflammatory reaction in response to tuberculosis infection. In our study, VEGF was present in the highest concentration among the cytokines measured. VEGF levels of BALF can be elevated in acute lung injury [24,25] and sarcoidosis [26]. Although VEGF is not a specific for tuberculosis, it could be a sensitive inflammatory marker.

IFN-γ is known to be a key cytokine in the host immune response to tuberculosis infection. Humans with IFN-γ receptor abnormalities and IFN-γ-deficient mice have increased susceptibility to *M. tuberculosis* infection [8,27]. Previous studies have reported high levels of IFN-γ in pleural effusions in TPE patients [11,28] and in BALF from patients with active pulmonary TB [29,30] or smear-negative active pulmonary TB [18]. An *in vitro* study has also found that the level of PPD-induced IFN-γ secretion by peripheral blood mononuclear cells (PBMC) from LTBI subjects was 20 times higher than the cut-off IFN-γ level (1.0 ng/ml) [31]. Another study found that IFN-γ mRNA expression levels in the lungs of latent TB mice were 7-times higher than that in the control mice [19]. In our study, we found that the levels of IFN-γ in BALF from TPE patients without pulmonary tuberculosis were 12-fold higher than that in the controls. We suggest that an increase in IFN-γ level in BALF indicates an inflammatory reaction in response to tuberculosis infection, similar to the IFN-γ test for LTBI.

It has been reported that cytokines associated with a T helper-2 (Th-2) response, IL-4, and IL-10 were higher than those associated with a Th-1 response in advanced TB patients; however, Th-2 responses were lower in early stages of TB infection [7]. *In vitro* stimulation of PBMC with *M. tuberculosis* antigens elicited Th-2 responses [7]. Cytokine mRNA expression levels in the lungs of mice indicated that Th-2 responses in LTBI mice were lower than Th-1 responses, and also IL-10 mRNA expression levels were lower than those of the control mice [19]. The present study showed that there was no difference in Th-2 responses, defined by IL-4 and IL-10 levels, between TPE patients and the controls. However, IL-10 was the only cytokine whose levels in TPE patients were lower than those in the controls. We suggest that Th-2 responses might have a minor role in the inflammatory reactions evident in the present study.

Increases in the CD4+/CD8+ ratio of T lymphocytes in BALF from active pulmonary TB patients, as compared to controls, have previously been reported [32,33]. Another study has demonstrated that the CD4+/CD8+ ratio was higher in susceptible TB mice [34]. In the present study, we showed that the CD4+/CD8+ ratio of T lymphocytes changes in patients without active TB infection.

Diagnosis of TPE based on ADA levels in pleural effusion may have a limitation. The positive predictive value (PPV) of pleural fluid ADA measurement in patients with TPE is depends on local prevalence of the disease [35]. The prevalence of TPE among all studied pleural effusions in our institution was about 23% between 2005 and 2010. This finding suggested that ADA has more than 80% of PPV in patients with TPE in our institution. In addition to high PPV value of ADA, all TPE patients in the present study were cured with anti-tuberculosis medication.

Another potential limitation of the present study is sensitivity of detecting active pulmonary tuberculosis by simple chest radiograph. High-resolution computed tomographies of patients with pleural tuberculosis were more sensitive than simple chest radiographs in detecting pulmonary tuberculosis activity [36]. We did not perform computed tomography of the study subjects. Although the present cases were all negative of BAL fluid MTB-PCR, AFB stain, and AFB culture, there could be a little possibility of active tuberculosis in the different lung region other than BAL area.

## Conclusions

We demonstrated significantly high levels of TNF-α, IFN-γ, and VEGF and an increased CD4+/CD8+ ratio of T lymphocytes in BALF from TPE patients without active pulmonary tuberculosis. IFN-γ, TNF-α, and VEGF would be an immune response markers of adjacent tissue in response to tuberculosis.

## Abbreviations

TB: Tuberculosis; TPE: Tuberculous pleural effusion; Th1: T helper-type 1; IFN-γ: Interferon-gamma; TNF-α: Tumor necrosis factor-alpha; VEGF: Vascular endothelial growth factor; BAL: Bronchoalveolar lavage; ADA: Adenosine deaminase; MTB-PCR: *M. tuberculosis* polymerase chain reaction; AFB: Acid-fast bacilli; BALF: Bronchoalveolar lavage fluid; ELF: Epithelial lining fluid; LTBI: Latent tuberculosis infection; Th-2: T helper-2.

## Competing interests

All authors have no competing interests.

## Authors’ contributions

DQ was responsible for data analysis, and for drafted this manuscript. WIC was responsible for carrying out the experiments, for data analysis, and for drafted this manuscript; KYK was responsible for the data analysis and interpretation. All authors contributed to the drafting and revisions of the manuscript.
